# Photodamage to the oxygen evolving complex of photosystem II by visible light

**DOI:** 10.1038/srep16363

**Published:** 2015-11-12

**Authors:** Alonso Zavafer, Mun Hon Cheah, Warwick Hillier, Wah Soon Chow, Shunichi Takahashi

**Affiliations:** 1 Research School of Biology, The Australian National University, Canberra, Australian Capital Territory, 2601 Australia

## Abstract

Light damages photosynthetic machinery, primarily photosystem II (PSII), and it results in photoinhibition. A new photodamage model, the two-step photodamage model, suggests that photodamage to PSII initially occurs at the oxygen evolving complex (OEC) by light energy absorbed by manganese and that the PSII reaction center is subsequently damaged by light energy absorbed by photosynthetic pigments due to the limitation of electrons to the PSII reaction center. However, it is still uncertain whether this model is applicable to photodamage to PSII under visible light as manganese absorbs visible light only weakly. In the present study, we identified the initial site of photodamage to PSII upon illumination of visible light using PSII membrane fragments isolated from spinach leaves. When PSII samples were exposed to visible light in the presence of an exogenous electron acceptor, both PSII total activity and the PSII reaction centre activity declined due to photodamage. The supplemental addition of an electron donor to the PSII reaction centre alleviated the decline of the reaction centre activity but not the PSII total activity upon the light exposure. Our results demonstrate that visible light damages OEC prior to photodamage to the PSII reaction center, consistent with two-step photodamage model.

Photosynthetic organisms including plants, algae and cyanobacteria use light energy to drive the oxygenic photosynthesis but paradoxically this process is accompanied by photodamage to photosystem II (PSII). Since photosynthetic electron flow starts from the reduction of plastoquinone by electrons released by oxidation of water at PSII, the accumulation of photodamaged PSII decreases photosynthetic activity. This phenomenon is referred to as photoinhibition^1^,^2^. To cope with photoinhibition, photosynthetic organisms have a PSII repair cycle that repairs photodamaged PSII^2^,^3^. When the rate of photodamage surpasses the rate of PSII repair, then net photoinhibition ensues. In addition, plants possess diverse photoprotection mechanisms that help avoid photoinhibition^4^. Therefore, photoinhibition happens only under unfavourable environmental conditions, e.g. environmental stress conditions^5^,^6^. Photoinhibition potentially happens in all photosynthetic organisms and severe photoinhibition may cause declines of growth and even mortality^7^.

The process of photodamage to PSII, which can be monitored in the absence of the PSII repair process, can be studied *in vitro* using isolated PSII complexes and thylakoid membranes and *in vivo* using antibiotics (chloramphenicol or lincomycin) that inhibit the PSII repair though inhibiting the synthesis of the D1 protein. The rate of photodamage to PSII in the absence of PSII repair is strongly related to the intensity of incident light^8^,^9^,^10^,^11^ and its wavelengths^12^,^13^,^14^,^15^,^16^, i.e. the photodamage rate coefficient is directly proportional to the intensity of light (with exceptions) and apparently faster under UV than visible light. It has been shown that the photodamage to PSII that happens under direct sunlight, in plants grown under sunlight, is largely associated with visible light since UV damage to PSII can be suppressed in such plants due to the accumulation of UV screening compounds^17^.

There are several hypothetical mechanisms associated with photodamage to PSII^5^,^18^,^19^,^20^,^21^. They are largely separated into two groups depending on the initial site of photodamage to PSII. In acceptor- and donor-side photoinhibition models, the photodamage to PSII initially happens at the site of PSII reaction centre which is associated with PSII electron transport^19^. The photodamage to PSII in these models is associated with light energy absorbed by photosynthetic pigments. In the two-step photodamage model, on the other hand, the photodamage to PSII initially happens at the water splitting site in the oxygen evolving complex (OEC) by light energy absorbed by manganese located in the OEC and secondary damage occurs at the PSII reaction centre because of light energy absorbed by photosynthetic pigments^12^,^13^. It is still uncertain which mechanism is mainly associated with photodamage to PSII in *in vivo* under visible light. Recent studies also suggest that both mechanisms may occur concurrently^22^,^23^,^24^,^25^.

The extent of photoinhibition is enhanced in conditions where the light energy absorbed by photosynthetic pigments exceeds its utilisation for photosynthesis, e.g., interruption of the Calvin-Benson cycle decreases the energy utilization capacity and accelerates photoinhibition. Therefore, it was widely assumed that excess light energy absorbed by antenna causes acceleration of photodamage to PSII through acceptor- or donor-side photoinhibition^26^. However, recent studies demonstrated that excess energy is not associated with the process of photodamage to PSII *per se*; excess energy causes photoinhibition through inhibiting the PSII repair^27^,^28^. Furthermore, the action spectrum of photodamage to PSII did not match with the light absorption spectrum of chlorophyll^12^,^13^,^16^. Moreover, reactive oxygen species produced under excessive light conditions cause photoinhibition through inhibiting the PSII repair but not through accelerating photodamage to PSII^10^,^29^,^30^,^31^. These results suggest that photodamage to PSII might not be associated with acceptor- or donor-side photoinhibition and not even with the light energy absorbed by photosynthetic pigments^5^,^6^,^20^,^32^.

In the two-step photodamage model, the initial step of photodamage to PSII is not associated with the excess energy and not even light energy absorbed by photosynthetic pigments^12^,^13^. Furthermore, the light absorption spectrum of model manganese compounds matches the action spectrum of photodamage to PSII^33^. Thus, the two-step photodamage model is consistent with recent experimental results. However, since manganese absorbs UV but less of visible light, it is still uncertain whether the two-step photodamage model is applicable to the mechanism of photodamage under visible light.

In this study, we examined the initial site of photodamage to PSII under visible light using PSII membrane fragments isolated from spinach leaves. We measured the PSII total activity (electron transfer from H_2_O via functional OEC and reaction center to an artificial electron acceptor) and the PSII reaction centre activity (electron transfer from an artificial electron donor via functional reaction center to an artificial electron acceptor) after PSII samples had been exposed to light in the presence of electron acceptor for PSII with or without electron donor for PSII. Our results demonstrate that an exogenous supply of electron donor for PSII alleviates the decline of PSII reaction center activity, but not PSII total activity, upon the visible light exposure. Our results show that photodamage to the PSII reaction center upon illumination with visible light is a secondary event following photodamage to the OEC. Here we propose that the two-step photodamage model is applicable to the photodamage to PSII upon illumination with visible light.

## Results

### Photodamage to PSII in PSII membrane fragments under visible light

**Figure 1:**
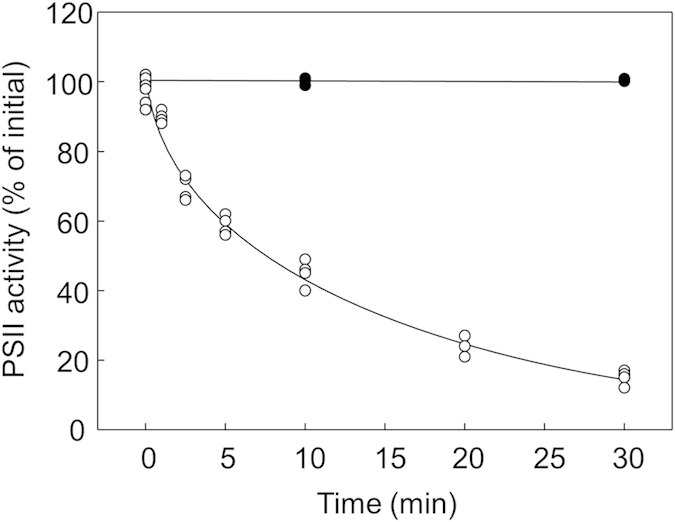
Decline of total PSII activity upon the light exposure in PSII enriched membrane particle. PSII samples were exposed to the light at 1000 μmol photons m^−2^ s^−1^ (open circles) with a halogen lamp or the darkness (closed circles) at 4 °C for maximum 30 min. Total PSII activity was then measured by monitoring the light dependent oxygen production.

In the present study, we used PSII membrane fragments isolated from spinach leaves to study the mechanisms of photodamage to PSII by visible light. Since the isolated PSII samples are unstable and lose their activity under ambient temperature, light treatments were carried out at a cold temperature (<4 °C). When PSII samples were exposed to visible light (photosynthetically active radiation at 1000 μmol photons m^−2^ s^−1^) for 30 min, the PSII activity measured by the production of oxygen declined to 10–20% of the initial activity (Fig. 1). The decline of PSII activity showed an approximately single exponential decay. Importantly, there was no decline of PSII activity in darkness (Fig. 1). These results demonstrate that the decline of PSII activity after the light exposure is solely due to the photodamage to PSII under these experimental conditions.

### No significant effect of exogenous electron acceptor on the photodamage to PSII

**Figure 2:**
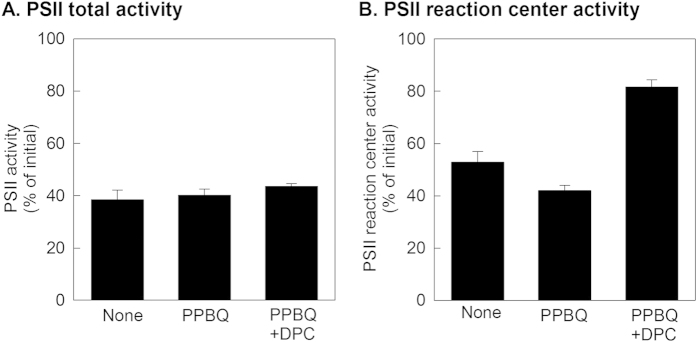
Declines of PSII total activity and PSII reaction center activity upon the visible light exposure. PSII samples were exposed to visible light at 1000 μmol photons m^−2^ s^−1^ with a xenon lamp at 4 °C for 5 min in the presence of electron acceptor PPBQ with or without electron donor DPC, or in their absence. PSII total activity (**A**) and PSII reaction center activity (**B**) were then measured by spectrophotometry. Values are mean of ±SD of 3 to 4 separate experiments.

We next monitored the decline of the PSII total activity upon the light exposure in the presence or absence of electron acceptor phenyl-p-benzoquinone (PPBQ). The PSII total activity was measured by spectrophotometry. When the PSII samples were exposed to light at 1,000 μmol photons m^−2^ s^−1^ for 5 min in the absence of PPBQ, the PSII total activity declined to 38% of initial activity (Fig. 2A). There was no significant effect of PPBQ on the decline of PSII total activity upon the light exposure; the activity declined to 40% of the initial activity. These results suggest that the electron flux at the acceptor side of PSII does not appear to influence the extent of photodamage to PSII.

### No significant effect of exogenous electron donor on the decline of PSII total activity

To examine the effect of electron donor on the extent of photodamage to PSII, PSII samples were exposed to light at 1,000 μmol photons m^−2^ s^−1^ for 5 min in the presence of the electron acceptor PPBQ with or without electron donor diphenylcarbazide (DPC). In the absence DPC, the total PSII activity declined to 40% of initial activity after the light exposure (Fig. 2A). There was no effect of DPC on the decline of PSII total activity in such a condition (Fig. 2A). These results demonstrate that the supplemental addition of an electron donor does not influence the extent of photodamage to PSII, suggesting that the electron flux at the donor side of PSII does not influence the extent of photodamage to PSII.

### Addition of exogenous electron donor alleviates the photodamage to PSII reaction center

When PSII samples were exposed to light at 1,000 μmol photons m^−2^ s^−1^ for 5 min in the absence of electron acceptor and donor, the PSII reaction center activity measured by spectrophotometry declined to 52% of the initial. Supplemental addition of electron acceptor PPBQ did not alleviate the decline of PSII reaction center activity upon the light exposure; the PSII reaction center activity declined to 42% of the initial. We then examined the effect of the electron donor DPC on the decline of the PSII reaction center activity upon the light exposure in condition where the electron acceptor PPBQ was present. Our results show that the decline of PSII reaction center activity upon the light exposure was alleviated by the presence of DPC; the PSII reaction center activity declined only to 82% of the initial activity. Thus, the presence of an electron donor did not influence the total activity of PSII (Fig. 2A) but significantly alleviated the photodamage to the PSII reaction center (Fig. 2B). This finding indicates that photodamage to the PSII reaction center under visible light, at least partially, happens after photodamage to the oxygen evolving complex due to limitation of electron supply.

### Photodamage to the oxygen evolving complex by light of different wavelengths

**Figure 3:**
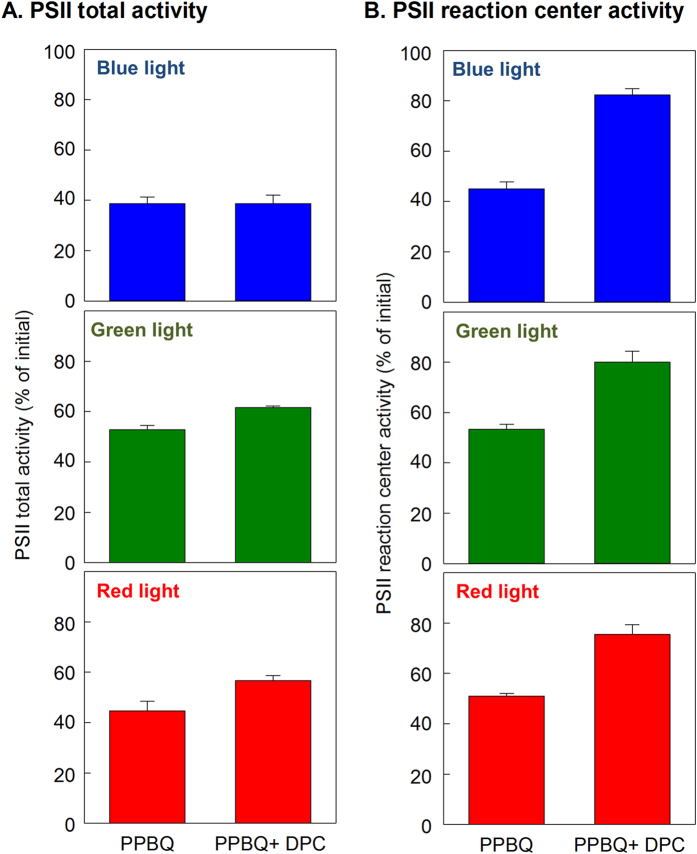
Decline of PSII total activity and PSII reaction center activity upon the exposure of different regions of visible light. PSII samples were exposed to different regions of visible light at 1000 μmol photons m^−2^ s^−1^ in the presence of electron acceptor PPBQ with or without electron donor DPC as follows: blue (400–530 nm; 5 min), green (530–590 nm; 30 min) and red (590–720 nm; 15 min). PSII total activity (**A**) and PSII reaction center activity (**B**) were then measured by spectrophotometry. Values are mean ± SD of four separate experiments.

To examine the effect of an electron donor on the total activity of PSII and PSII reaction center activity, PSII samples were exposed to different wavelength regions of light in the presence of an exogenous electron acceptor PPBQ, with or without an exogenous donor DPC. Then, PSII total activity and reaction centre activity were spectrophotometrically measured. The samples were exposed to light at 1000 μmol photons m^−2^ s^−1^ as follows: blue (400–530 nm; 5 min), green (530–590 nm; 30 min) and red (590–720 nm; 15 min). PSII total activity declined to 39%, 52% and 45% of initial when PSII samples were exposed to blue, green and, red lights, respectively, in the absence of DPC (Fig. 3). The decline of PSII total activity upon green-light and red-light exposure was slightly alleviated by the supplemental addition of DPC. There was no effect of DPC on the decline of PSII total activity upon blue-light exposure. The PSII reaction centre activity declined to 45%, 53%, and 51% of the initial after the exposure of blue, green and, red light, respectively, in the absence of DPC. The decline of PSII reaction centre activity upon the light exposure was apparently alleviated by the supplemental addition of DPC and the activity declined only to 82%, 80%, 75% of the initial after exposure to blue, green, and red lights, respectively. These results demonstrated that any regions of visible light spectrum can cause photodamage to the OEC. Photodamage to the PSII reaction centre is likely due to limitation of electron supply from the OEC.

## Discussion

### Mechanism of visible light-associated photodamage to PSII

**Figure 4:**
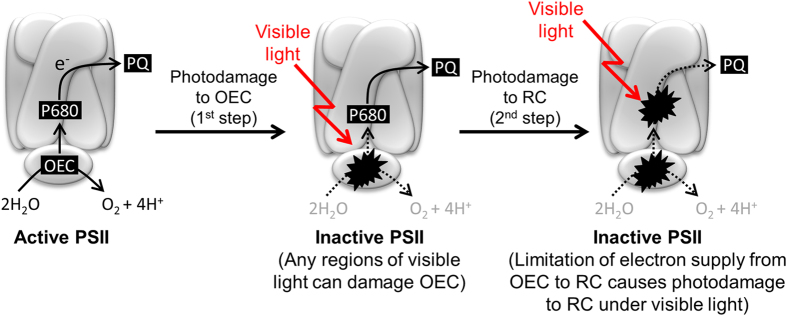
Proposed sequence of photodamage to PSII by visible light. Visible light initially damages the oxygen evolving complex (OEC). Then, PSII reaction center (RC) is secondary damaged by visible light due to limitation of electron supply from water to the PSII reaction center. PQ, plastoquinone.

In the present study, we examined the effect of an exogenous electron donor on the extent of loss of total PSII activity and PSII reaction centre activity upon the light exposure. Our results demonstrated that the presence of exogenous electron donor during light exposure to PSII alleviated photodamage to the PSII reaction centre (Fig. 2B) but not photodamage to PSII as a whole (Fig. 2A) in the presence of electron acceptor. These results indicate that photodamage to the OEC is the primary event and photodamage to the PSII reaction centre is a secondary event that occurs due to the limitation of electron supply from water to the PSII reaction centre (Fig. 4). Importantly, a similar effect of the exogenous electron donor on the extent of photodamage to PSII and PSII reaction center was shown under different regions of visible light spectrum (blue, green and red) (Fig. 3). This finding indicates that any regions of visible light can cause photodamage to the OEC while photodamage to the PSII reaction center is a secondary event. This finding is consistent with the proposed two-step photodamage model where the primary site of photodamage is at the OEC while damage to the reaction centre is secondary^12^,^13^. However, it is noted that addition of an exogenous electron donor did not completely negate photodamage to the PSII reaction centre. Thus, we still cannot completely exclude the possibility of photodamage to PSII as a whole by visible light initially occurring at the PSII reaction centre through other mechanisms.

In the two-step photodamage model, primary photodamage to OEC is hypothesised to be associated with light absorbed by manganese in the OEC. This model is supported by results showing that the light absorption spectrum of model manganese is similar to the action spectrum of photodamage to PSII and that manganese is released from PSII after photodamage to OEC by UV and blue light^12^. Since light absorption spectra of model manganese compounds show very low light absorbance at visible light wavelengths^12^, it is controversial whether any regions of visible light can damage OEC. However, our results clearly demonstrate that any region of visible light can initially damage the OEC and secondarily the reaction centre (Fig. 4). This result provides a possibility that the manganese in the OEC absorbs less visible light but still in quantities sufficient to damage OEC. However, we must stress that the present data do not allow us to test the validity of the hypothesis that direct absorption of light by manganese in the OEC is the cause of photodamage to the OEC.

### Limitation of electron acceptor and donor of PSII does not accelerate photodamage to PSII

Previously, photodamage to PSII was proposed to be due to light energy excessively absorbed by photosynthetic pigments, leading to the development of acceptor- and donor- side photoinhibition models. In these models, photodamage to PSII is associated with limitation of electron acceptor of PSII (acceptor-side photoinhibition) or limitation of electron donor to the excited P680 (donor-side photoinhibition) both due to excessive excitation of P680 by light absorbed by photosynthetic pigments^19^,^26^. However, recent studies have proposed that excess energy accelerates photoinhibition through inhibition of the PSII repair but not due to acceleration of photodamage to PSII^27^,^28^. Furthermore, in our present study, there was no significant effect of electron acceptor or donor on the extent of photodamage to PSII (Fig. 2). This result is incompatible with the acceptor and donor side photoinhibition hypothesis, suggesting that photodamage to PSII is less associated with light energy excessively absorbed by photosynthetic pigments. Consistent with this new hypothesis, impairment of photoprotection mechanisms associated with dissipating excessively absorbed light energy, such as photorespiratory pathway^34^, thermal energy dissipation^35^ and ROS scavenging^10^,^29^, had no influence on the process of photodamage. All these photoprotection mechanisms have been shown to avoid inhibition of the repair of photodamaged PSII under excessive light conditions^4^.

### Is photodamage to PSII related to light energy absorbed by photosynthetic pigments?

In the present study, PSII samples were more sensitive to photodamage under blue and red lights than green light. This is in agreement to an earlier report of action spectrum in visible light^14^ and examined in high resolution in the red region^36^,^37^. Since chlorophylls have light absorption peaks at blue and red wavelengths, light energy absorbed by chlorophylls seems likely to be associated with photodamage to PSII. These results supports the mechanism whereby light energy absorbed by photosynthetic pigments causes photodamage to PSII^19^,^26^. However, other reports of action spectra of photodamage to PSII showed a strong peak at UV wavelengths toward blue light and no significant peak in red light regions^12^,^15^. Furthermore, under sunlight, PSII seems less sensitive to blue and red than green in intact leaves^16^. Therefore, it is still unclear whether photodamage to PSII under visible light is associated with light energy absorbed by photosynthetic pigments.

### Photodamage to PSII under strong sunlight

In plants grown under sunlight, the photodamage to PSII happens under sunlight is largely associated with visible light^17^. Previously, photodamage to PSII was assumed to be attributed to light energy excessively absorbed by photosynthetic pigments. Therefore, acceptor- or donor-side photoinhibition model was applied to describe the mechanism of photodamage to PSII under sunlight. However, our results demonstrated that the two-step photodamage model is more applicable to describe the mechanism of photodamage to PSII upon the visible light illumination (Fig. 4). Therefore, photodamage to PSII under the sunlight might be more related light energy absorbed by OEC rather than photosynthetic pigments. Indeed, it has been demonstrated that the photodamage to PSII happens under sunlight is mostly related to yellow light, which is less absorbed by photosynthetic pigments, in the visible light regions^16^. However, the excessive light energy absorbed by photosynthetic pigments inhibits the repair of photodamage PSII^27^,^28^. Thus, based on the two step photodamage model, photosynthetic organisms exposed to direct (strong) sunlight, e.g., leaves on the upper canopy, are at the risk of photoinhibition due to photodamage to PSII by light absorbed by OEC and inhibition of the PSII repair upon excess light energy absorbed by photosynthetic pigments.

## Material and Methods

### Sample preparation

PSII enriched membranes were prepared from fresh spinach leaves purchased from the market following a previous method^38^. Then the sample was solubilized in standard buffer (400 mM sucrose, 25 mM MES-NaOH, 15 mM NaCl, 5 mM MgCl_2_, pH 6.5), flash frozen in liquid nitrogen (LN_2_) and stored at −80 °C until use. Before light exposure, the sample was unfrozen and resuspended in Buffer A (40 mM Mes buffer, pH 6.5, 40 mM sucrose). In order to decrease the sucrose content, the sample was centrifuged at 24000 × *g* for 5 minutes and suspended in fresh Buffer A. The chlorophyll content was measured^39^ and fixed to 150 μg of chlorophyll mL^−1^. The sample was kept in darkness at 4 °C at all times unless otherwise stated.

### Photodamage treatments

PSII samples (150 μg of chlorophyll mL^−1^) were exposed to light at 4 °C in the presence or absence of electron donor DPC (10 μM) and electron acceptor PPBQ (0.6 mM). The thickness for sample illumination was adjusted to 2.5 mm in all cases. A halogen lamp projector was used as a visible light source (for emission spectrum see Fig. S1). A xenon light source (MAX-303, Asahi Spectra Co. Ltd. Japan) with a combination of short and long-pass filters were used as light source in the blue (400–530 nm), green (530–590 nm) and red (590–720 nm) regions of the visible spectra (Fig. S1). All photodamage treatments were carried out at 4 °C.

### PSII total activity and PSII reaction centre activity assay

After photodamage treatments, PSII samples (150 μg chlorophyll mL^−1^) were collected by centrifugation (16000 × *g*, 10 min), resuspended in fresh Buffer A and adjusted to 7.5 μg of chlorophyll mL^−1^. PSII reaction center activity and PSII total activity were measured spectrophotometrically in the presence of the artificial electron acceptor 2,6-dichlorophenol indophenol (DCPIP) (1 mM) at 25 °C with or without artificial electron donor DPC (100 μM), respectively, using a UV-vis spectrophotometer (Cary 300, Varian). The assay was illuminated using 430 nm LED (2400 μmol of photons m^−2^ s^−1^) and the activity was monitored by absorption changes at 600 nm due to reduction of DCPIP. The detector was protected by a narrowband filter centred at 600 nm to ensure that the PMT received only the monochromatic light transmitted by the sample.

## Additional Information

**How to cite this article**: Zavafer, A. *et al.* Photodamage to the oxygen evolving complex of photosystem II by visible light. *Sci. Rep.* **5**, 16363; doi: 10.1038/srep16363 (2015).

## Supplementary Material

Supplementary Data
