# Supplementary material for: Photodamage to the oxygen evolving complex of photosystem II by visible light
Source: Sci Rep. 2015 Nov 12;5:16363. doi: 10.1038/srep16363 (PMC4642293; doi:10.1038/srep16363)

**Photodamage to the oxygen evolving complex of Photosystem II by visible light**

**Alonso Zavafer**, Mun Hon Cheah, Warwick Hillier, Wah Soon Chow, Shunichi Takahashi


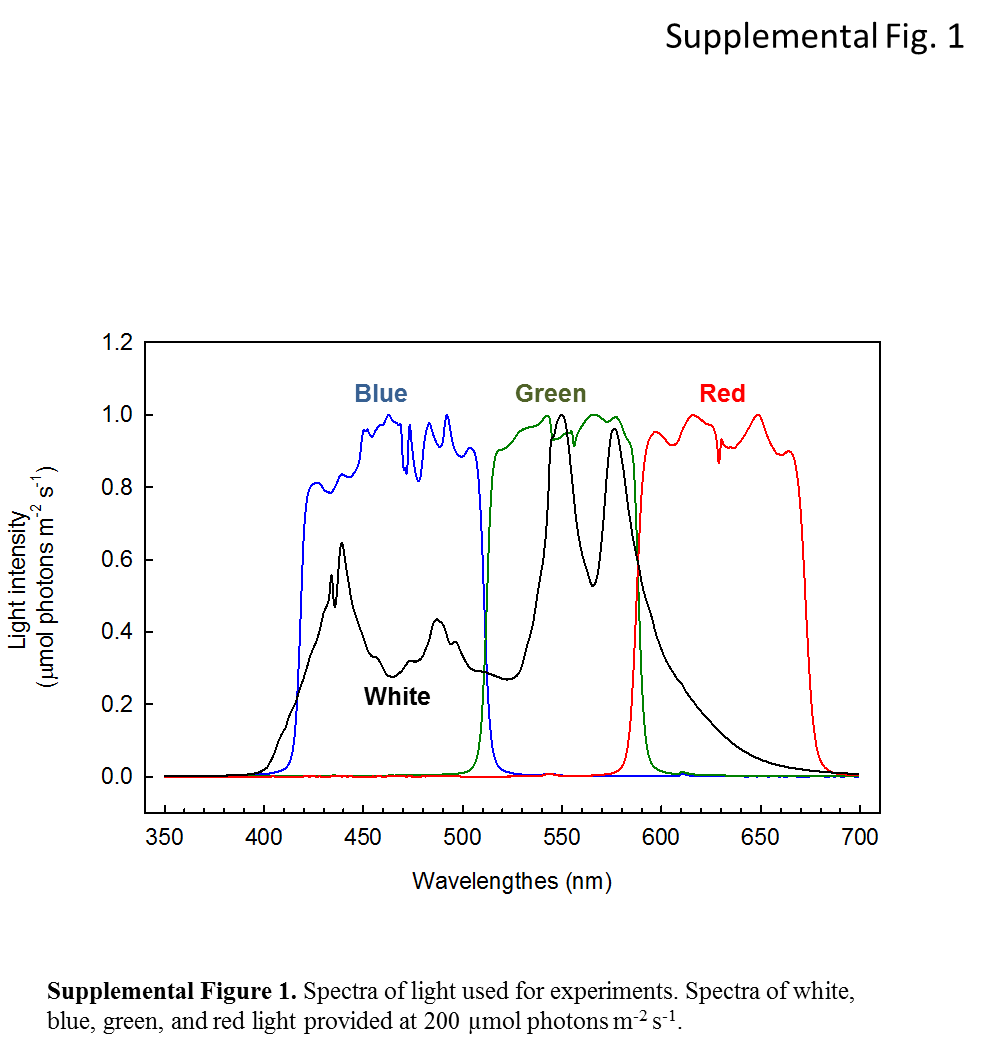

Supplement: Supplementary Data [file srep16363-s1.doc]
